# Cardiovascular Diseases in COPD: From Diagnosis and Prevalence to Therapy

**DOI:** 10.3390/life13061299

**Published:** 2023-05-31

**Authors:** Anastasia Papaporfyriou, Konstantinos Bartziokas, Daniela Gompelmann, Marco Idzko, Evangelia Fouka, Stavrina Zaneli, Petros Bakakos, Stelios Loukides, Andriana I. Papaioannou

**Affiliations:** 1 Division of Pulmonology, Department of Internal Medicine II, Medical University of Vienna, 1090 Vienna, Austria; dranastp@gmail.com (A.P.); daniela.gompelmann@meduniwien.ac.at (D.G.); marco.idzko@meduniwien.ac.at (M.I.); 2 Independent Researcher, 42132 Trikala, Greece; bartziokas@gmail.com; 3 General Hospital G. Papanikolaou, Pulmonary Department of Aristotle University of Thessaloniki, 57010 Thessaloniki, Greece; evafouka@gmail.com; 4 1st University Department of Respiratory Medicine, “Sotiria” Chest Hospital, National and Kapodistrian University of Athens, 11527 Athens, Greece; stavzaneli@gmail.com (S.Z.); petros44@hotmail.com (P.B.); 5 2nd Respiratory Medicine Department, “Attikon” University Hospital, National and Kapodistrian University of Athens, 12462 Athens, Greece; loukstel@med.uoa.gr

**Keywords:** COPD, cardiovascular comorbidities, COPD exacerbation, COPD treatment

## Abstract

Chronic obstructive pulmonary disease (COPD) is considered one of the leading causes of mortality. Cardiovascular comorbidities are diagnosed often in COPD patients, not only because of the common risk factors these two diseases share, but also because of the systemic inflammation which characterizes COPD and has deleterious effects in the cardiovascular system. The comorbid cardiovascular diseases in COPD result in several difficulties in the holistic treatment of these patients and affect outcomes such as morbidity and mortality. Several studies have reported that mortality from cardiovascular causes is common among COPD patients, while the risk for acute cardiovascular events increases during COPD exacerbations and remains high for a long time even after recovery. In this review, we focus on the prevalence of cardiovascular comorbidities in COPD patients, presenting the evidence regarding the interaction of the pathophysiological pathways which characterize each disease. Furthermore, we summarize information regarding the effects of cardiovascular treatment on COPD outcomes and vice versa. Finally, we present the current evidence regarding the impact of cardiovascular comorbidities on exacerbations, quality of life and survival of COPD patients.

## 1. Introduction

Chronic obstructive pulmonary disease (COPD) is a major cause of morbidity and mortality in patients older than 40 years of age, and it is projected to become the fourth leading cause of premature death by 2040 [1]. In 2023, COPD has been redefined as “heterogeneous lung condition characterized by chronic respiratory symptoms (dyspnea, cough, sputum production and/or exacerbations) due to abnormalities of the airways (bronchitis, bronchiolitis) and/or alveoli (emphysema) that cause persistent, often progressive, airflow obstruction” [2]. A new definition has been stated for AECOPD (Acute Exacerbation of COPD) too: “an event characterized by dyspnea and/or cough and sputum that worsen over ≤14 days, which may be accompanied by tachypnea and/or tachycardia and is often associated with increased local and systemic inflammation caused by airway infection, pollution, or other insult to the airways” [3]. Both definitions focus mostly on these diseases’ proper characteristics instead of on their epidemiology, risk factors, cause and diagnostic criteria. However, COPD has a considerable impact on patients’ quality of life, while exacerbations increase direct health care cost (pharmacotherapy and/or hospitalization) and indirect costs (mainly related to a reduction of working days due to dyspnea and functional deterioration) [4,5]. Smoking history and advanced age of COPD patients increase the risk for various additional disorders, known as comorbidities, which affect outcomes and complicate treatment [6]. It has been reported that approximately 86–98% of COPD patients suffer from at least one comorbid condition [7]. A previous study has shown that comorbidities are responsible for approximately one-third of deaths in COPD patients [8], while the risk of death is higher in patients with multiple comorbidities [9]. Accordingly, management of comorbidities and standard treatment, including smoking cessation, pharmacotherapy, pulmonary rehabilitation, and immunization, are critical components of contemporary COPD care [10].

One of the most prevalent comorbidities of COPD is cardiovascular disease (CVD) [11], which constitutes the leading cause of mortality worldwide, affecting approximately 85 million people in Europe [12]. The Global Initiative has highlighted Coexisting COPD and CVD for Chronic Obstructive Lung Disease (GOLD) as being highly important, since CVD is responsible for over half of hospitalizations and deaths in COPD patients [13]. COPD and CVD are two entities that share similar pathophysiological mechanisms, and frequently occur in the same individual, while medications used to treat CVD can either benefit or harm COPD patients and vice versa. In the absence of definite recommendations on the treatment of patients with COPD and CVD, this narrative review aims to provide recent advances in better understanding of the mechanisms leading to the coexistence of these diseases and to provide information on the effect of specific treatments for either disease on the other, both during stability and during exacerbations.

## 2. Methods

We searched Medline (via PubMed) up to February 2023 to identify studies relevant to this review. The combination of the following keywords was used as search terms: “Cardiovascular diseases in COPD”, “Prevalence”, “Treatment”, “Survival and Quality of Life”. In addition, the reference lists of the retrieved articles were further searched to identify their relevance to the concept of this review.

## 3. Frequency and Type of Cardiovascular Comorbidities in COPD

The frequency of CVD in COPD varies throughout studies, depending mainly on disease severity, number of comorbidities, cohorts recruited from primary or secondary care, methodology, and adjustment for confounding factors. However, it seems that COPD patients are two to five fold more likely to develop cardiovascular diseases, including coronary artery disease (CAD), cardiac dysrhythmia, heart failure (HF), and pulmonary and peripheral vascular disease (PVD), compared to the general population [14,15]. It is important to point out that this increase in the prevalence of CVD in COPD patients occurs even after adjustment for age, smoking habit, and other risk factors [15].

### 3.1. Heart Failure (HF)

The prevalence of HF in COPD ranges from 7% to 42% and is significantly higher compared to the non-COPD population [16]. At the same time, HF-related hospitalizations are higher in patients with COPD (1807 vs. 352 per 100,000 person-years for COPD and non-COPD patients, respectively). Furthermore, the prevalence of HF is approximately 30% in patients hospitalized for COPD exacerbations, reaching almost 75% in those receiving mechanical ventilation [17,18,19]. Large epidemiological studies report that COPD patients with concomitant HF have a worse prognosis in terms of acute exacerbations, hospitalizations, and mortality [20,21,22]. In the same context, HF is the most frequent cause of hospital readmission and worsens the short and long-term prognosis of patients hospitalized for COPD exacerbation [17]. Conversely, the prevalence of COPD in patients with HF lies in the range of 13.0–39.0% [23], while the presence of COPD worsens the prognosis of patients hospitalized due to HF [24].

### 3.2. Ischemic Heart Disease (IHD) and Coronary Artery Disease (CAD)

The frequency of IHD among stable COPD patients seems to range between 7.1% and 33% [7,25,26] and reaches 17–22% in patients admitted to the hospital for COPD exacerbations [27]. Recently, it has been reported that COPD patients with clinical manifestation of IHD have significantly decreased exercise capacity (according to the six-minute walking test (6MWT)), more severe dyspnea (according to the modified medical research council (mMRC) dyspnea scale), decreased body mass index, more significant airflow obstruction and a worse quality of life [28]. Furthermore, COPD patients with concomitant IHD have a twofold increased chance of being hospitalized [29] and greater 3-month mortality [17], while IHD is one of the leading causes of death for this group of patients [30].

10.0% to 17.0% of patients with a confirmed diagnosis of COPD have experienced an acute myocardial infarction, and COPD roughly doubles the risk of myocardial infarction, a clinical outcome associated with a worse prognosis [31,32].

Although the data vary across different studies, 20.0–60.0% of COPD patients suffer from CAD [14,33]. A systematic analysis of the Global Burden of Disease Study conducted in 2017 found that the most common cause for early mortality in COPD was CAD, accounting for more than one million deaths worldwide [34].

Peripheral artery disease (PAD) is a condition similar to CAD, characterized by atherosclerotic plaques leading to occlusion of the arteries of the lower limbs. In a large cohort of COPD patients, 8.8% were diagnosed with PAD, compared to 1.8% in subjects without COPD (control group), while PAD was associated with a clinically significant decline in functional ability and health status [35]. As expected, the prevalence of PAD in COPD patients increases with the severity of airway obstruction and is estimated at between 13% and 16% [17,27]. In a cohort of 3742 patients who underwent peripheral vascular interventions or lower extremity bypass for PAD, the coexistence of COPD was linked with a higher readmission rate [36] whereas, in a prospective cohort study of 170 outpatients with stable COPD, an ankle-brachial index of <1 was found to be an independent factor for 4-year mortality [37].

### 3.3. Arrhythmia

Arrhythmia prevalence estimates may be confounded by asymptomatic and paroxysmal AF (atrial fibrillation), as the diagnosis may be limited to self-reporting [38]. Nevertheless, it has been shown that the prevalence of arrhythmia in patients with stable COPD ranges between 5% and 15% reaching 20–30% in patients with severe disease [39]. Two systematic analyses reported that the prevalence of arrhythmias in COPD was 5% to 29% and increased in patients with more severe lung function impairment [14,33]. AF represents the most frequently encountered supraventricular arrhythmia in COPD, with an estimated prevalence among stable COPD patients of between 4.7% and 15.0%, while rates range from 20.0% to 30.0% in patients with very severe disease [40]. In patients hospitalized for COPD exacerbation, atrial fibrillation was observed in 21%, while arrhythmia appeared in 27% [27]. During COPD exacerbations, AF appears more frequently when PaCO2 (partial pressure of carbon dioxide) and pulmonary artery systolic pressure are higher [41]. COPD patients with AF have a worse health-related quality of life (HRQoL), while mortality rates among patients hospitalized for COPD exacerbations increase when AF is present [42,43]. Furthermore, it seems that COPD exacerbations may trigger AF, while AF per se is also a risk factor for COPD exacerbations [44]. On the other hand, the presence of COPD seems to increase the risk of hospital admission due to AF [45].

### 3.4. Systemic Hypertension and Stroke

Systemic hypertension is the most frequent comorbidity in COPD patients, and even though it does not affect the progression of COPD per se, it is a vascular risk factor for the development of CVD [46]. Consequently, in COPD patients, systemic hypertension can induce the onset of heart failure and ischemic heart disease, aggravating the prognosis of COPD [47]. Its prevalence in COPD patients ranges between 28.5% and 64.7% [48,49], but the rates may vary [14,33]. Therefore, all patients with COPD and systemic hypertension must have a thorough assessment to identify the risk factors for both diseases. Finally, COPD is known to increase the risk of stroke, which appears in approximately 10% of patients with stable COPD and approximately 20% of those hospitalized with COPD exacerbation [50].

## 4. Interplay between CVD and COPD

Over the past decade, there has been a significant improvement in our understanding of the relationship between COPD and different types of CVD. Although exposure to deleterious gases is the common risk factor for both diseases, as shown in the Rotterdam study [51], it seems that their coexistence is associated with more complex inflammatory mechanisms, especially in patients under 65 years of age [52]. Thus, although smoking contributes to the occurrence of stroke [51], heart failure, and coronary artery disease, being a crucial risk factor for atherosclerotic disease and atherosclerotic plaque rupture [53], the interaction between CVD and COPD involves numerous additional biological processes, including hypoxia, systemic inflammation, endothelial dysfunction, increased platelet reactivity, and arterial stiffness [54,55].

Systemic inflammation [56] is implicated in the onset, development, and rupture of atherosclerotic lesions [57], playing a crucial role in the pathogenesis of CVD [56,57] and HF [58]. There is evidence that inflammation is also linked to the development of arrhythmias, and CVD prognosis has been shown to be correlated with the levels of inflammatory markers, such as intercellular cell adhesion molecule 1 (ICAM-1), interleukin-6, C-reactive protein, and serum amyloid-A. [59] Blood inflammatory markers, such as CRP, are increased in COPD patients, whereas individuals with CVD have higher blood concentrations of fibrinogen, interleukin-6, interleukin-8, and other inflammatory markers than those without comorbidities [60]. Patients suffering from both CVD and COPD express elevated levels of interleukin-6, CRP, and fibrinogen. Similarly, inflammatory biomarkers appear to be increased during and immediately after an exacerbation of COPD, when the risk of cardiovascular events and mortality is higher [61]. There is a significantly higher risk of CVD events within 30 days after COPD hospitalization and exacerbation, which seems to be related to the elevated levels of circulating proinflammatory biomarkers known to play a significant role in the comorbidity of COPD and CVD [62,63].

As cross-sectional data exhibit that elevated arterial stiffness is independently related to COPD, the growing understanding of the increased cardiovascular risk associated with COPD has led to a request for respiratory physicians to evaluate arterial pulse wave velocity in standard practice [64]. Arterial stiffness may be caused due to the imbalance between matrix metalloproteases and is related to elastin degradation, leading to emphysema in the lung and the systemic disruption of elastin in the vasculature [65]. Arterial stiffness plays a key role among the different surrogate measures of cardiovascular risk and is a powerful independent predictor of cardiovascular events beyond the traditional cardiovascular risk factors, considered as a surrogate marker of coronary, cerebrovascular, and peripheral artery disease [52]. Although Sabit and co-workers initially reported a correlation between arterial stiffness, the degree of airway obstruction, and systemic inflammation in COPD patients [66], it is unclear whether arterial stiffness has a causative relationship with CVD and COPD or only represents a complication of these concomitant conditions.

The primary cause of atherosclerosis is vascular endothelial dysfunction, which refers to the imbalance of vasodilator and vasoconstrictor substances produced by endothelial cells, which in turn, is a critical factor in the pathogenesis of CVD [67]. Vascular endothelial injury recognized in COPD is the result of increased inflammatory response and oxidative stress, contributing to the development of CVD in these patients and thus dramatically increasing the risk of cardiovascular disorders such as atherosclerosis, myocardial infarction, and stroke [68]. Some studies reveal that patients with COPD have impaired endothelial-dependent and endothelium-independent vascular function compared to controls [69]. In contrast, other studies suggest that, when inflammatory mediators are removed, the integrity of the vascular endothelium can be restored, and the incidence of CVD is reduced [70]. The higher risk of cardiovascular disease in COPD patients may be explained by this interrelation between systemic inflammation and impaired systemic vascular function.

Patients with COPD often experience hypoxia, which is either sustained in those with severe disease or intermittent, appearing during exercise or at the time of exacerbation. Hypoxia induces CVD risk via numerous mechanisms, including elevated systemic inflammation and oxidative and hemodynamic stress [55]. Hypoxia stimulates the release of cell adhesion molecules on the vascular endothelium, allowing circulating leukocytes to adhere to the vascular intima and increasing the risk of atherosclerosis through promoting proinflammatory cytokines and oxidative stress [71]. Moreover, carotid-femoral pulse wave velocity (PWV) has been significantly associated with the partial pressure of oxygen in the arterial blood (PaO2) in COPD, indicating that hypoxemia may lead to increased arterial stiffness [64]. In addition, studies have shown that static hyperinflation in COPD has the most robust relationship with heart size and that airway obstruction is associated with decreasing heart size [72]. Apart from being evident in severe emphysema, a decreased heart size has been shown to have a strong correlation with hyperinflation across the whole severity spectrum of COPD leading to hypoxemia [73]. Moreover, it is well known that patients with COPD may experience airflow restriction, which can cause static and dynamic chest hyperinflation. This can make breathing work harder and put more strain on the respiratory muscles, leading to increased intrathoracic pressure. High intrathoracic pressure inhibits venous return to the right ventricle by compressing the intrathoracic big veins and the right atrium. While the left ventricle is primarily affected by bigger pressure changes and the systemic circulation is placed in compartments of different pressures, the right ventricular function is largely influenced by preload due to the ventricular filling [74]. Additionally, due to increased lung volume and increased peripheral vascular resistance (PVR), which is a result of dynamic hyperinflation, the intrathoracic pressure increases during exhalation, decreasing the right ventricular (RV) preload and increasing the RV afterload [75]. Recent data from Lukacsovits et al. has shown that the intrathoracic pressure during exercise in COPD patients has significantly greater swing than healthy individuals, thereby affecting stroke volume and cardiac output [76].

Patients with COPD and IC/TLC (Inspiratory Capacity/Total Lung Capacity) of ≤0.25 have a defective global right ventricular function and left ventricular diastolic filling, independently associated with reduced exercise tolerance [77]. Pulmonary vascular remodeling and vasoconstriction are also a consequence of chronic hypoxemia in stable COPD patients, which leads to right-ventricular diastolic dysfunction and thus contributes to CVD risk [78]. This is accomplished through rising pulmonary vascular resistance, which results in the displacement of the interventricular septum to the left ventricle, which in turn may cause impairment of ventricular filling, stroke volume, and cardiac output [79].

## 5. Effect of Treatment for CVD on COPD and Vice Versa

Although available data is contradictory, there are some studies supporting that the use of cardiovascular drugs reduces mortality in patients with COPD-heart failure overlap [80] and decelerates disease progression, although they might be underused in COPD patients [81]. Routine medications for CVD include β-blockers, angiotensin-converting enzyme inhibitors (ACEIs), angiotensin II receptor blockers (ARBs), antiplatelets and statins. The German COPD cohort COSYCONET (COPD and Systemic Consequences—Comorbidities Network), a prospective, multicenter cohort study including patients with stable COPD, reported that patients with COPD and heart failure appear less likely to receive appropriate therapy for heart failure and thus experience a worse prognosis [82].

### 5.1. B- Blockers

The safety of b-blockers in COPD patients is debatable, since these drugs can potentially cause bronchoconstriction and decrease the effectiveness of both short and long-acting 2-agonists [83]. However, in 2017, a large prospective study, including patients from the COPDGene cohort, has shown that, regardless of the severity of the airway obstruction, the use of b-blockers is linked to a considerable reduction in COPD exacerbations, while a systematic review, which included randomized controlled trials [84], concluded that cardio-selective b-blockers do not have serious adverse respiratory reactions and can also reduce the risk of COPD exacerbations. 

On the other hand, a meta-analysis comparing multiple studies concluded that the use of b-blockers was related to a larger FEV_1_ decline in patients with higher lung function at baseline, while a prospective, randomized controlled trial on the use of metoprolol in patients with moderate to severe COPD was discontinued after b-blocker was associated with a higher risk of hospitalizations due to exacerbation [85]. 

The 2016 European Society of Cardiology guidelines advocate their use in patients with COPD and CVD [86]. A meta-analysis conducted in 2020 found that b-blockers are safe and reduce all-cause mortality (including in-hospital mortality) in patients with COPD, while selective b-blockers may even reduce the acute incidence of COPD exacerbations [87]. Finally, they can withstand the elevated heart rate caused by bronchodilators and do not interfere with their effectiveness [87]. In conclusion, owing to their conflicting pharmacological effects, the use of beta-blockers in COPD has been debatable.

### 5.2. Angiotensin-Converting Enzyme Inhibitors (ACEIs) and Angiotensin II Receptor Blockers (ARBs)

ACEIs and ARBs are two common renin-angiotensin-aldosterone system inhibitors used to treat cardiovascular disease CVD, but there is limited data on their use in COPD patients. Data from a large population-based trial, the Multi-Ethnic Study of Atherosclerosis Lung Study, revealed that both ACE inhibitors and ARBs were related to slower progression of emphysema, particularly among former smokers [88]. Similarly, another study conducted in Taiwan concluded that risks of pneumonia and severe exacerbations were significantly lower in COPD patients receiving ARBs than in those receiving ACEIs [89]. Furthermore, irbesartan, an angiotensin receptor blocker, was well tolerated in patients with stage III and IV COPD, raising the possibility that patients with chronic obstructive pulmonary disease may obtain unexpectedly favorable effects from taking these classes of medication [90]. The recently published PARADIGM-HF (Prospective Comparison of Angiotensin Receptor Blocker-Neprilysin Inhibitor With Angiotensin-Converting Enzyme Inhibitor to Determine Impact on Global Mortality and Morbidity in Heart Failure) trial demonstrated that the usage of RAAS inhibitors in individuals with heart failure and COPD was consistent with those without COPD, indicating that these drugs did not cause more side effects in patients with COPD [91].

### 5.3. Diuretics

Loop diuretics, thiazide diuretics, and potassium-sparing diuretics are the three primary categories of diuretics widely used for the management of patients with heart failure. Diuretics can improve pulmonary ventilation, decrease pulmonary congestion and edema, and increase lung compliance, which is extremely helpful for COPD patients [92,93]. In a comparative effectiveness study among patients with concomitant hypertension and COPD requiring two antihypertensive agents, combination therapy with a thiazide diuretic was related to a significantly lower risk of hospitalization for congestive heart failure among patients without a history of congestive heart failure [94]. The side effects arising from the use of diuretics are well known and include hypokalemia, elevated pO2, metabolic alkalosis, and decreased cardiac output [95]. Consequently, to obtain personalized treatment, the type and dosage of diuretics should be decided with regard to the underlying condition.

### 5.4. Antiplatelets

Antiplatelet therapy contributes significantly to the reduction of all-cause mortality in COPD patients [96]. The role of antiplatelets in the management of COPD and CVD has been investigated in several studies. According to a prospective national study, antiplatelet medications increased COPD patients’ survival rates, perhaps due to a systemic antithrombotic effect [97], while a post-hoc analysis of the PLATO trial (Platelet Inhibition and Patient Outcomes) [98] showed that, without raising the overall rate of major bleeding incidents, antiplatelet treatment significantly decreased the absolute risk of ischemic events in COPD patients. COPD is associated with a high prothrombotic setting, indicating that anticoagulation therapy may impact CVD morbidity and mortality in these patients in addition to antiplatelet therapy [99]. Regarding the use of aspirin in COPD patients, it has not been found to alter lung function parameters [100]. However, observational studies recommend that it is associated with fewer acute exacerbations of COPD, reduced progression of emphysema, lower mortality, and better quality of life [96,101,102].

### 5.5. Statins

Statins, i.e., 3-hydroxy-3-methylglutaryl coenzyme A (HMG-CoA) reductase inhibitors, decrease not only total cholesterol, LDL, and triglyceride levels but also exhibit pleiotropic pharmacological actions, such as antioxidant, anti-inflammatory, antithrombotic and immunomodulatory effects [103]. Since the early 2000s, treatment with statins has been associated with improved survival in COPD [104], although they may be only partially beneficial, as they do not reduce pulmonary exacerbations nor improve lung function [105]. Although observational studies which included COPD patients who used statins for cardiovascular and metabolic purposes have shown an association between statin use and improved outcomes (such as reduced exacerbations and mortality) [106], this effect of statins in exacerbations was not proved in patients with COPD who had no metabolic or cardiovascular indication for statin treatment [107]. Finally, it is worth mentioning that statins reduce the risk of pulmonary hypertension in COPD, an effect that seems to be dose- and time-dependent [108].

### 5.6. COPD Medication

Medications currently used for COPD treatment may also have an impact on the outcome of cardiovascular diseases. Even though tachyarrhythmias and hypokalemia are well-known side effects of bronchodilators, they have a fundamental role in COPD treatment [109]. Numerous studies have shown that the severity of COPD and disease exacerbations are associated with increased cardiovascular events and worse mortality from CVD [110,111]. Bronchodilators are classes of drugs that increase FEV_1_ and improve other spiro-metric parameters, contribute to the reduction of dynamic hyperinflation at rest and during exercise, thus improving exercise performance, and reduce the number of exacerbations [46]. The extent of these changes suggests that these drugs may have a beneficial effect on CVD outcomes. Generally, little research has been done on the cardiovascular risk related to the LAMA/LABA combinations. An extensive network meta-analysis involving 23 studies found that LAMA/LABA combination therapy had comparable effects to either monotherapy on safety outcomes [112]. Moreover, a meta-analysis contrasting dual bronchodilation with monotherapy showed a greater improvement in FEV_1_ with a slight difference in health status with LAMA/LABA combination compared to monotherapy, with no escalation in cardiovascular risk [113]. On the contrary, a real-world primary care database revealed an increased risk of developing heart failure one year after switching from single to dual bronchodilator medication, but with no evident higher risk of acute myocardial infarction, stroke, or arrhythmia [114]. Moreover, in recent years, data indicate that long-acting dual bronchodilators have been related to an increased risk of cardiac events in comparison with monotherapy [115] whereas, compared with ICS/LABA, LAMA/LABA combination or triple therapy escalates cardiovascular risk in COPD patients [116].

Concerning inhaled corticosteroids (ICS), an increase in their administered dose probably reduces CV events and leads to an improvement in lung function, reduction of exacerbations of COPD, and potential reduction of all-cause mortality [117,118]. Nevertheless, in the SUMMIT study, which recruited a large cohort of COPD patients with CVD or major risk factors, inhaled corticosteroids showed no effect on cardiovascular events [110].

## 6. COPD Exacerbations Course and Treatment in Patients with CVDs

AECOPD is characterized by increased dyspnea and/or cough and sputum, which may be accompanied by tachypnea and/or tachycardia and is often associated with increased local and systemic inflammation [46]. However, COPD exacerbations are not only presented with worsening respiratory symptoms but are also tumultuous situations leading to aggravated adverse events [119]. The risk of CV events is increased during COPD exacerbations, especially in patients with CVD or CV risk factors, as a result of lung inflammation and oxidative stress [54,60]. Several studies have tried to uncover the causative relationship between COPD exacerbations and CV events. Donalson et al. (2010) showed a 2.2-fold higher possibility of MI within a brief 5-day window and a 1.3-fold higher possibility of stroke within 49 days after COPD exacerbation [120]. Reilev et al. (2019) found, instead, a 4-fold risk for cardiovascular death and higher risks for MI and stroke (incidence rate ratio (IRR) = 2.58, 95% CI = 2.26–2.95 and (IRR = 1.97, 95% CI = 1.66–2.33 respectively) during the 4-week period after the onset of an AECOPD and a stronger association in the subgroup analysis for the older age groups [121].

A post-hoc analysis of UPLIFT confirmed that cardiovascular events are the most common adverse events during and after AECOPD [122]. Moreover, it has been reported that there is a sixfold higher risk of stroke after a severe AECOPD compared to stable disease, probably due to the presence of hypoxia and acute inflammation during AECOPD [51]. Rothnie et al. described that, during AECOPD, patients at greater risk for cardiovascular complications (mainly non-STEMI MI) had experienced fewer exacerbations in the previous year than those with more significant lung function impairment. [123] Regarding the time of onset of a CV event, Goto et al. showed that adverse cardiovascular events occur both in short- (30-day) as well as long-term periods (1 year) after AECOPD [124], while Wang et al. showed that the risk of cardiovascular adverse events after hospitalization for AECOPD is high for at least 90 days and is associated with mortality [125].

### 6.1. Pathophysiology

Patho-physiologically, the biomarker profile or inflammatory pathway of COPD exacerbation that can cause a CV event remains unclear. It has been hypothesized that higher levels of Troponin T [126] and renal endothelin-1 production [127] or increased circulating fibrinogen levels [128] and endothelial dysfunction [129] during AECOPD are associated with increased cardiovascular risk. AECOPD are provoked mainly by viral and/or bacterial airway infection, which can also trigger systemic inflammatory processes, with elevated production of acute phase biomarkers such as plasma fibrinogen and C-reactive protein (CRP) [130]. There is a direct connection between plasma fibrinogen and thromboembolic events, while CRP can lead to the adhesion of leukocytes to arterial endothelium [131]. A recent meta-analysis of Theodorakopoulou et al. supports the dysfunction of endothelium-dependent, but mostly endothelium-independent, vasodilation of the branchial artery during AECOPD [132]. The hypoxemia caused by AECOPD is yet another possible explanation for CV events [124], since it increases pulmonary artery pressures leading to right heart strain, cardiac remodeling, and AF, or through endothelial dysfunction to thromboembolic events [133,134].

The study of Labonté et al. has shown that Club Cell protein 16 (CC-16) and V-rel avian reticulo-endotheliosis viral oncogene homolog B (RelB) were inversely associated with arterial stiffness during AECOPD and up to 6 months after the exacerbation [135]. CC-16 is known for its connection with lung function and COPD progression [136,137], while in the study mentioned above its levels could predict exacerbation frequency [135]. RelB, an anti-inflammatory cytokine against cigarette smoke-induced inflammation, belongs to the NFkB family and seems to have a negative connection with systolic blood pressure during COPD exacerbation [138]. Furthermore, the levels of CD34+ cells, influenced by inflammation during an exacerbation, correlate with cardiac parameters (NT-proBNP plasma levels, LVEF, PASP, and resting heart rate) that predict CV events during AECOPD [139]. 

The causal nexus between AECOPD and CV events could be explained through the abrupt rise in airway resistance during an exacerbation that substantially reduces lung emptying and expiratory flow [60]. As a result, during an exacerbation, patients often breathe quickly and shallowly, creating an endless loop whereby lung emptying time decreases, and dynamic hyperinflation (DH) rises. As a consequence of DH, pulmonary hypertension worsened (intra-alveolar vessel compression), right ventricular pre-load reduced, and, sometimes, left ventricular afterload increased, establishing favorable circumstances for CV events [140].

### 6.2. Treatment

Although short courses of OCS (up to 5 Days) can rarely induce dyslipidemia, hypertension, or hyperglycemia, COPD patients with frequent exacerbations could be affected by the side effects of systemic steroid therapy. Additionally, these patients are treated with ICS, which can eventually result in undesirable side effects even when prescribed in moderate doses. Dyslipidemia, hypertension, and hyperglycemia are key factors in a variety of CV events [141]. Furthermore, the intensified use of bronchodilators during an AECOPD could increase heart rate and lead to arrhythmias and myocardial ischemia in patients with CVD [142]. On the other hand, a combination of SABA with LAMA in a model of lung hyperinflation during exercise resulted in faster QT kinetics and more pronounced improvements in microvascular oxygen delivery compared to placebo [143]. The use of specific antibiotics, such as clarithromycin, in patients with AECOPD may be linked to excessive cardiovascular events that persist beyond the prescribed term [144], though according to the cohort study of Berni et al., it is more likely the site of infection rather than the use of antibiotics that increases the risk for CV events [145]. Overall, it is impossible to completely rule out the impact of time-dependent confounding brought on by these medications [121].

## 7. Life Expectancy and Quality of Life

In COPD patients, cardiovascular disease continues to be one of the leading causes of death [9,146]. Indeed, mortality linked to cardiac illness is higher than death related to respiratory failure in individuals with moderate COPD [147]. In the study of Mannino et al., cardiovascular diseases and diabetes mellitus predicted higher mortality than other comorbidities in patients with COPD [9]. Among patients with moderate to severe COPD, in the TORCH trial, death-rate due to CV events was 26% [148], and in a large Danish meta-analysis was about the same at 24.8% [121], whereas in the UPLIFT trial this was estimated at 18.8% and in a post-hoc analysis of SUMMIT trial was even lower at 5.7% [63,122]. Among the most common CV comorbidities, Divo et al., in their multicenter RCT, found that Atrial Fibrillation, Congestive Heart Failure, and Coronary Artery Disease were independently associated with COPD mortality [149]. On the other hand, patients with myocardial infarction have a higher mortality rate when COPD co-exists [150] and a higher incidence of cardiovascular-related mortality, myocardial infarction, and arrhythmias is linked to decreased pulmonary function in COPD patients [151,152,153,154].

The combination of COPD and CVD also significantly influences patients’ Quality of Life. According to the National Health and Nutrition Examination Survey, Chronic Heart Failure was associated with worse self-related health (OR 3.07, 95% CI 1.69–5.58) in COPD patients, independent of age, gender, and race [155]. Inversely, in a systematic review that examines the impact of non-cardiovascular comorbidities on the Quality of Life of patients with chronic heart failure, a statistically significant negative association was found between respiratory diseases, mainly COPD, and Quality of Life [156].

## 8. Conclusions

**Figure 1:**
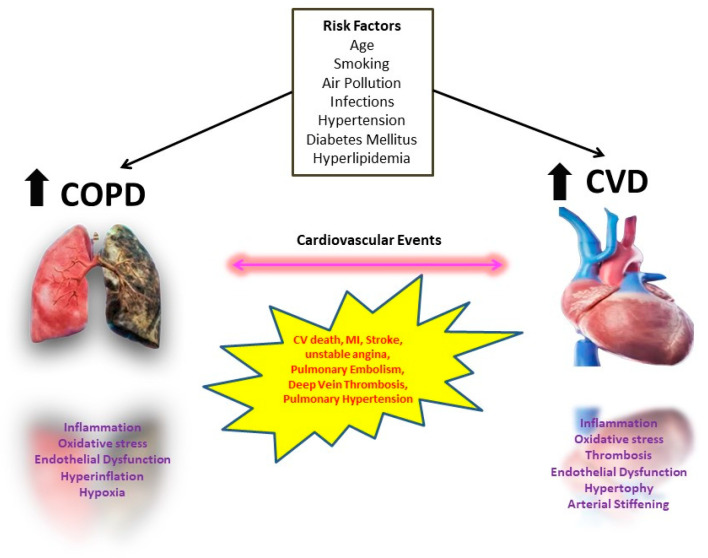
Interplay between COPD and Cardiovascular diseases.

In conclusion, as the presence of CVDs has a significant impact on the prognosis of COPD, the diagnosis and appropriate treatment of these comorbidities is a crucial step in the management of COPD. (Figure 1 summarizes the interactions between cardiovascular events and COPD). Due to the pathophysiological and inflammatory linkages between COPD and CVDs, all physicians must evaluate COPD patients for cardiac comorbidities, both under stable conditions and during exacerbations.

## Data Availability

Not applicable.
